# Total nasal resistance among Sasang constitutional types: a population-based study in Korea

**DOI:** 10.1186/1472-6882-13-302

**Published:** 2013-11-04

**Authors:** Dae Wui Yoon, Seung Ku Lee, Hyeryeon Yi, Jeong Hwa Hong, Miyazaki Soichiro, Si Woo Lee, Jong Yeol Kim, Chol Shin

**Affiliations:** 1 Institute of Human Genomic Study, College of Medicine, Korea University Ansan Hospital, Ansan, Republic of Korea; 2 Department of Nursing, Korea Nazarene University, Cheonan, Republic of Korea; 3 Department of Control and Measurement Engineering, Korea University, Seoul, Republic of Korea; 4 Department of Sleep Medicine, Shiga University of Medical Science, Shiga, Japan; 5 Constitutional Medicine and Diagnosis Research Group, Korea Institute of Oriental Medicine, Daejeon, Republic of Korea; 6 Department of Pulmonary, Sleep and Critical Care Medicine, College of Medicine, Korea University Ansan Hospital, Ansan, Republic of Korea

**Keywords:** Sasang constitutional types, Total nasal resistance, Active anterior rhinomanometry, Transnasal pressure, Tae-eum type, So-eum type, So-yang type

## Abstract

**Background:**

There have been many attempts to find an objective phenotype by Sasang constitutional types (SCTs) on an anatomical, physiological, and psychological basis, but there has been no research on total nasal resistance (TNR) among SCTs.

**Methods:**

We assessed the value of the TNR in the SCTs classified by an integrated diagnostic model. Included in the study were 1,346 individuals (701 males, 645 females) who participated in the Korean Genome and Epidemiology Study (KoGES). The TNR was measured by active anterior rhinomanometry (AAR) at transnasal pressures of 100 and 150 Pascal (Pa).

**Results:**

The average TNR was 0.186 ± 0.004 Pa/cm^3^/second at 100 Pa in the Tae-eum (TE), 0.193 ± 0.007 in the So-eum (SE), and 0.208 ± 0.005 in the So-yang (SY) types. Under condition of 150 Pa the TE type had a TNR value of 0.217 ± 0.004, the SE type was 0.230 ± 0.008, and the SY type was 0.243 ± 0.005. Higher values of TNR were more likely to be reported in the SY type at 100 Pa and 150 Pa. In the stratified analysis by sex, the SY type in males and females tended to have higher TNR value than the TE and SE types at transnasal pressure of both 100 Pa and 150 Pa.

**Conclusions:**

These results provide new approaches to understand the functional characteristics among the SCTs in terms of nasal physiology. Further studies are required to clarify contributing factors for such a difference.

## Background

Sasang constitutional medicine (SCM) is a branch of Korea traditional medicine. It was first introduced by Jema Lee (AD 1837–1900) a Korean physician a century ago. According to the SCM theory, humans can be classified into four distinct constitutions: Tae-yang (TY), Tae-eum (TE), So-yang (SY), and So-eum (SE) types, in terms of different efficacy and sensitivity to some types of herbs and medicines, disease susceptibility, difference in functional activity of internal organs, physical traits, and psychological characteristics [1]. Based on the concept that Sasang constitution types (SCTs) have a distinct susceptibility to disease or drug response, it is possible to offer the safe and effective use of medicine or treatment according to individual traits, called personalized medicine [1].

Nasal airway resistance accounts for up to 60% of total airway resistance [2]. Several kinds of environmental (e.g., alcohol, drug, exercise, and air temperature) and intrinsic factors (e.g., inflammation in nose, ventilation, posture) may affect nasal resistance. Exercise, rebreathing, and erect posture result in reduction of nasal resistance, whereas infective or allergic rhinitis, hyperventilation, alcohol, and cold air increase nasal resistance [3].

Increased nasal resistance elevates negative inspiratory pressure, which in turn make the upper airway vulnerable to the tendency to collapse during sleep, hence increasing the possibility of having sleep-disordered breathing [4,5], as well as negatively affecting the quality of sleep [6,7].

Rhinomanometry is a functional test of nasal aerodynamics used to objectively determine nasal obstruction and understand nasal physiology. It was introduced in the 1950s and early 1960s [8]. It measures transnasal airflow and the pressure gradient between the nasopharynx and in front of the nose simultaneously. It is possible to calculate the value of nasal resistance from these data [8]. Currently, three methods of rhinomanometry have been used: anterior rhinomanometry, posterior rhinomanometry, and postnasal rhinomanometry. Among them, active anterior rhinomanometry (AAR) is the most common rhinomanometric method for assessment of nasal patency and resistance [9], recommended by the Standardization Committee on Objective Assessment of the Nasal Airway as the method of choice for measuring nasal ventilation [10].

Since development of several tools which can objectively classify and predict SCTs, there have been many attempts to find specific associations between chronic disease and SCTs and physiological features typical of SCTs. As a result of those efforts, it was found that SCTs can act as a risk factor for metabolic syndrome [11], insulin resistance [12], hypertension (HTN) [13], and diabetes mellitus (DM) [14], but to our knowledge no studies have reported total nasal resistance among SCTs. This study, therefore, assessed the total nasal resistance among SCTs by AAR through a population-based study in Korea.

## Methods

### Subjects

**Figure 1:**
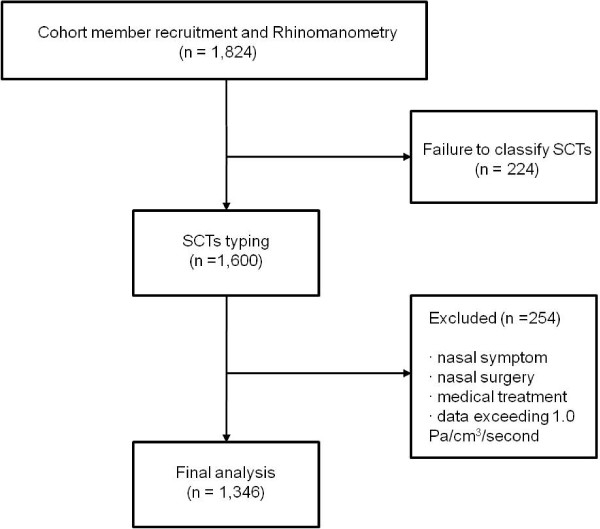
Subject selection for analysis of total nasal resistance among SCTs.

The subjects were selected from cohort members participated in the Korean Genome and Epidemiology Study (KoGES). The cohort members consist of the 5,020 Koreans (2,523 men and 2,497 female) aged 40 to 69 years from 2001 who participated in a comprehensive health examination and on-site interviews at Korea University Ansan Hospital. They were followed up biannually and examined for anthropometric evaluation, demographic information, blood pressure, spirometry, and routine blood tests. Each participant signed written informed consent form before the health examination. Participants were asked if they had symptoms of nasal disturbance such as nasal obstruction, sneezing, and rhinorrhea or upper airway infections and any previous nasal surgery or treatment. Among 1,824 individuals who enrolled in the cohort from 2010 to 2011 and had undergone rhinomanometry from 2007 to 2009, 1,600 subjects were classified as the SCTs using an integrated diagnostic model developed by Do et al. [15]. 254 subjects with at least one nasal symptom or histories of nasal surgery or medical treatment or TNR value exceeding 1.0 Pa/cm^3^/second were excluded. Finally, 1,346 subjects were employed for the analysis (Figure 1). The protocols of this study were approved by the Institutional Review Board at Korea University Ansan Hospital. There was no one from our sample who was classified as the TY type, consistent with previous reports. Therefore, only data from the TE, SE, and SY constitutions was evaluated in this study.

### Classification of SCT

Each constitutional type was classified by an integrated diagnostic model developed by Do et al. [15]. The newly developed diagnostic model integrates four individual quantitative data such as facial, body shape, voice analysis, and questionnaire responses into a single model. Although QSCCII has been frequently used to classify the SCT, the diagnostic power of the model is superior that of QSCCII due to the following reasons: First, the accuracy of the current diagnostic method is higher than that of QSCCII (64% in males and 55.2% in females vs. 51% in total); Second, several variables were used for the integrated diagnostic method and were combined to classify SCTs, while only one variable such as questionnaire information was used for QSCCII; Third, quantitative data used for the development of the integrated diagnostic method was acquired from many Oriental medical clinics, while data for QSCCII was collected from a single site [15,16].

The facial images of subjects were taken with a digital camera, and variables expressing facial points and contours were automatically extracted *via* image processing techniques. Measurements for analysis to express body shape characteristics include the following eight items: forehead circumference, neck circumference, axillary circumference, chest circumference, rib circumference, waist circumference, pelvic circumference, and hip circumference. Among initially extracted 222 features by two voice programs, Hidden Markov Model Toolkit (HTK) and Praat, 88 features were selected by a genetic algorithm-based feature selection technique and employed for voice analysis. 67 multiple-choice questions to represent personality characteristics and physiological symptoms were included in the questionnaire.

### Rhinomanometry

Active anterior rhinomanometry (AAR) is the most frequently used rhinomanometric tools for assessment of nasal patency and resistance in research and clinical fields. All measurements were performed by a skilled technician using a rhinorheograph MRP-3100 (Nihon Kohden Co., Tokyo, Japan) as previously described [17]. The subjects were tested in a sitting position in a temperature and humidity-controlled room. To measure AAR one nasal cavity is obstructed while the other cavity is left open for flow measurements. A nasal adapter was placed inside the obstructed nostril. The value of unilateral nasal resistance measurements may show daily variation even in the same subjects due to alternation between nostrils, the so called nasal cycle [18], thus nasal resistances were measured for both nostrils at a transnasal pressure of 100 and 150 Pascal (Pa). The resistance of inspiratory airflow at each nostril was represented as a transnasal differential pressure (Pa) divided by nasal airflow (V), as follows:

R = ΔP/V

R: resistance (Pa/cm^3^/second)

ΔP: transnasal differential pressure (Pa)

V: nasal airflow (cm^3^/second).

The AAR for both nostrils was calculated to acquire TNR values according to the Standardization Committee on Objective Assessment of the Nasal Airway recommendations [9]. The TNR was represented in Pa/cm^3^/second at a transnasal pressure of 100 Pa or 150 Pa. Data exceeding 1.0 Pa/cm^3^/second were excluded since they are likely to have structural or mucosal abnormality which has not being ruled out by questionnaire.

### Health examination, questionnaire on life style, and biochemical measurements

Body mass index (BMI) was calculated as weight in kilograms divided by height in meters squared measured to the nearest 0.1 cm or 0.1 kg. Waist circumference (cm) was measured at the narrowest point between the lower rib and the iliac crest. Blood pressure (BP) was measured in a sitting position with a mercury sphygmomanometer on the non-dominant arm. The information on smoking status (never, former, or current), alcohol consumption (g/day), and exercise (30 min/2 times/week) was asked in the questionnaire. Biochemical measurements for plasma fasting glucose and insulin, total cholesterol, triglyceride (TG), and high density lipoprotein (HDL) cholesterol were conducted in the Seoul Clinical Laboratories (Seoul, Korea).

### Definition of hypertension and diabetes mellitus

Hypertension was defined as systolic/diastolic blood pressure more than 130/90 mmHg or use of antihypertensive medications. Diabetes mellitus was defined as fasting glucose levels over 100 mg/dL or use of antihyperglycemic agents.

### Statistical analysis

The TNRs and other data are expressed as mean ± standard deviation. One-way analysis of variance (ANOVA) analysis and χ^2^ for continuous variables and categorical variables were conducted to evaluate significant differences of the means among SCTs. Bonferroni’s *post hoc* test was performed for multiple comparisons. Multivariate linear regression models were used to examine the relationships between SCTs and TNR value. Age, weight, height, and smoking status were adjusted in the models. P trends across categories were calculated in the regression models. Statistical analysis was performed with SAS version 9.1 (SAS Institute, Cary, NC, USA). Null hypotheses of no difference were rejected if p-values were less than .05.

## Results

### General characteristics of participants

The 1,346 participants (male = 701, female = 645) who underwent rhinomanometry were included for the analysis. The general characteristics of the participants according to the SCT are summarized in Table 1. The TE type was older, heavier, and had a higher proportion of females and former smokers than those of the other types (*P* <0.0001, each). The TE type consumed about twice as much alcohol as the SE and SY types (*P* = 0.0003). In the blood chemical analysis, the levels of fasting glucose, fasting insulin, TG, HDL cholesterol were significantly higher in the TE type as compared to any other groups. The total cholesterol levels did not differ by the SCTs. The TE group had higher SBP and DBP, and higher prevalence of hypertension and DM, compared to the SE and SY types.

**Table 1:** General characteristics of participants according to Sasang constitutional types

**Variable**	**TE**	**SE**	**SY**	**P value**
No. of cases	709	193	444	
Age	56.4 ± 9.3^1)a^	52.8 ± 8.7	53.9 ± 9.4	< 0.0001
Female, n (%)	408 (57.6)^b^	95 (49.2)^c^	142 (32)	< 0.0001
Smoking, n (%)				
Never	346 (48.8)	110 (57.0)	290 (65.3)	< 0.0001
Former	243 (34.3)^bc^	48 (24.9)	107 (24.1)	
Current	120 (16.9)	35 (18.1)	47 (10.6)	
Exercise^2)^, n (%)	265 (40.5)	67 (40.1)	179 (44.8)	0.356
Alcohol consumption (g/day)	9.5 ± 17.6^bd^	5.6 ± 20.2	5.5 ± 17.0	< 0.001
BMI (kg/m^2^)	26.6 ± 2.3^a^	21.3 ± 1.7^c^	22.9 ± 2.0	< 0.0001
WHR (Waist-to-hip ratio)	0.9 ± 0.1^a^	0.8 ± 0.1	0.8 ± 0.1	< 0.0001
Fasting glucose (mg/dL)	104.5 ± 28.9^a^	90.9 ± 13.5	93.8 ± 14.2	< 0.0001
Fasting insulin (uU/mL)	10.1 ± 8.5^a^	6.8 ± 2.4	7.6 ± 2.7	< 0.0001
Total cholesterol (mg/dL)	195.7 ± 36.8^a^	201.4 ± 32.7	198.7 ± 34.4	0.094
Triglyceride (mg/dL)	161.4 ± 119.5	116.6 ± 61.4	123.3 ± 74.5	< 0.0001
HDL cholesterol (mg/dL)	45.6 ± 10.7^a^	53.4 ± 15.1	52.7 ± 14.2	< 0.0001
SBP (mmHg)	119.2 ± 32.5^b^	112.0 ± 34.1^e^	112.9 ± 33.2	0.001
DBP (mmHg)	79.3 ± 31.7	75.6 ± 34.4	75.6 ± 32.0	0.107
HTN, n (%)	307 (43.3)^a^	34 (17.6)	105 (23.7)	< 0.0001
DM, n (%)	180 (25.6)^a^	12 (6.4)^e^	62 (14.5)	< 0.0001

*Abbreviation*: *TE* Tae-eum type, *SE* So-eum type, *SY* So-yang type, *BMI* body mass index, *HDL* high density lipoprotein, *SBP* systolic blood pressure, *DBP* diastolic blood pressure, *HTN* hypertension, *DM* diabetes mellitus.

^1)^Values are mean ± SD.

^2)^30 min/2 times/week.

^a^*P* <0.0001 vs. SE and SY types.

^b^*P* <0.05 vs. SE type.

^c^*P* <0.0001 vs. SY type.

^d^*P* <0.001 vs. SY type.

^e^*P* <0.01 vs. SY type.

### Total nasal resistance among SCTs

TNR measurements are represented in Table 2. The average TNR was 0.186 ± 0.004 Pa/cm^3^/second at 100 Pa in the TE type, 0.193 ± 0.007 in the SE type, and 0.208 ± 0.005 in the SY types. After multivariate adjustment for age, sex, weight, height, and smoking status, a high value of TNR was more likely to be reported in the SY type at 100 Pa (*P* for trend < 0.01). Under condition of 150 Pa the TE type had a TNR value of 0.217 ± 0.004, the SE type was 0.230 ± 0.008, and the SY type was 0.243 ± 0.005. Consistent with the results in 100 Pa, the SY type was more likely to have a high TNR value at 150 Pa (*P* for trend < 0.01). In the stratified analysis by sex, the SY type in females tended to have higher TNR value than the TE and SE types at transnasal pressure of both 100 Pa and 150 Pa (*P* for trend = 0.05 and 0.03, respectively). We also found that a high TNR value was more likely to be reported in the SY type in males at 100 Pa and 150 Pa (*P* for trend = 0.02, respectively).

**Table 2:** TNR values according to Sasang constitutional types

		**TE**	**SE**	**SY**	
Total	No. of subjects	79	193	444	P for trend^b^
	TNR (Pa/cm3/sec)				
	100 Pa	0.186 ± 0.004^a^	0.193 ± 0.007	0.208 ± 0.005	<0.01
	150 Pa	0.217 ± 0.004	0.230 ± 0.008	0.243 ± 0.005	<0.01
Female	No. of subjects	301	98	302	P for trend^c^
	TNR (Pa/cm3/sec)				
	100 Pa	0.193 ± 0.006	0.202 ± 0.010	0.212 ± 0.006	0.05
	150 Pa	0.223 ± 0.007	0.238 ± 0.011	0.248 ± 0.006	0.03
Male	No. of subjects	408	95	142	P for trend^c^
	TNR (Pa/cm3/sec)				
	100 Pa	0.179 ± 0.005	0.190 ± 0.011	0.203 ± 0.007	0.02
	150 Pa	0.210 ± 0.005	0.228 ± 0.012	0.235 ± 0.008	0.02

*Abbreviation*: *TE* Tae-eum type, *SE* So-eum type, *SY* So-yang type, *TNR* total nasal resistance, *Pa* pascal.

^a^Data are presented as means ± SD.

^b^Adjusted for age, sex, weight, height, and smoking status.

^c^Adjusted for age, weight, height, and smoking status.

## Discussion

To the best of our knowledge, this is the first large population-based study to determine the values for nasal resistance among SCTs. The present study revealed that the SY type tended to have higher TNR value than the TE and SE types. There are several studies regarding the values of healthy population for nasal resistance, including European, East Asian, and African [19-22]. Although it is difficult to directly compare the value of nasal resistance among different races due to different age of subjects and sample sizes in the studies, Europeans have the highest value of nasal resistance, whereas Africans have the lowest value. In Korea, the mean TNR of normal subjects having a mean age of 53.4 ± 9.2 has been reported as 0.19 ± 0.08 and 0.22 ± 0.09 Pa/cm^3^/second at 100 Pa and 150 Pa, respectively [17]. Thus, although higher value of TNR was found in the SY type as compared to other types, the value of all SCTs is within a normal range at both 100 Pa and 150 Pa.

The reason TNR was higher in the SY type is unknown. Facial anatomical structures such as dimensions of the external nose, nasal width, and nostril diameters and difference in nasal mucosal blood flow could be contributing factors although it requires further studies [18,21].

The best strength of our study is the large sample size and the study design of a general population-based study. The limitation of the present study is that the age of subjects was limited to middle age. Most subjects were more than 50 years old (mean age 55.1 ± 9.3 yrs), and there were no subjects in the range between 20 to 49 years, the relatively young population. That is because the analysis was done with subjects who were followed between 2010 and 2011, while the beginning of KoGES study was in 2001 with participants aged 40 to 69 years old. Given that nasal resistance is highest in infants and declines with age [17,23], the possibilities of bias from age distribution cannot be ruled out.

## Conclusions

The value of TNR differs according to SCTs, and the SY type had a higher value of TNR as compared to those of the TE and SE types. These results may contribute to the understanding of nasal physiology among SCTs in terms of nasal patency and resistances. Further studies will be needed to clarify what factors determine such differences in TNR among SCTs.

## Abbreviations

SCTs: Sasang constitutional types; SCM: Sasang constitutional medicine; TNR: Total nasal resistance; AAR: Active anterior rhinomanometry; TY: Tae-yang type; TE: Tae-eum type; SE: So-eum type; SY: So-yang type; HTN: Hypertension; DM: Diabetes mellitus; KoGES: Korean genome and epidemiology study; HTK: Hidden Markov model toolkit; Pa: Pascal; BMI: Body mass index; TG: Triglyceride; HDL: High density lipoprotein; ANOVA: Analysis of variance.

## Competing interests

The authors declare that they have no financial competing interests.

## Authors’ contributions

DWY and SKL: conceived the idea, designed the study, analyzed data, and wrote the paper. HRY: collected and analyzed the data. JHH: interpreted and revised the data. SM: interpreted and revised the data. SWL: revised the manuscript and designed the study. JYK: revised the manuscript and designed the study. CS: designed and approved the study. All authors contributed to manuscript preparations and approved the final manuscript.

## Pre-publication history

The pre-publication history for this paper can be accessed here:

http://www.biomedcentral.com/1472-6882/13/302/prepub
